# Supplementary material for: Attract the best: The attraction effect as an effective strategy to enhance healthy choices
Source: PLoS One. 2021 Nov 4;16(11):e0259521. doi: 10.1371/journal.pone.0259521 (PMC8568290; doi:10.1371/journal.pone.0259521)
Supplement: S1 Table — (PDF) [file pone.0259521.s001.pdf]

S1 Table. Choice sets with mean perceived level of attractiveness and healthiness.

Unhealthy choice set			
	Target	*Competitor*	*Decoy*
Products	M&M's	Bonbons	Sweets
Attractiveness	80.64	83.96	54.08
Healthiness	19.16	13.16	17.84
Healthy choice set			
	Target	*Competitor*	*Decoy*
Products	Snack Tomatoes	Unsalted Cashews	Granola Cookies
Attractiveness	58.36	60.40	36.32
Healthiness	86.48	67.04	66.88
Mixed choice set			
	Target	*Competitor*	*Decoy*
Products	White Grapes	Chocolate Chip Cookies	Carrots
Attractiveness	76.52	77.88	56.00
Healthiness	78.16	18.04	86.00
