# Supplementary material for: Attract the best: The attraction effect as an effective strategy to enhance healthy choices
Source: PLoS One. 2021 Nov 4;16(11):e0259521. doi: 10.1371/journal.pone.0259521 (PMC8568290; doi:10.1371/journal.pone.0259521)
Supplement: S2 Table — (PDF) [file pone.0259521.s002.pdf]

S2. Table. Choice sets main experiment.

	Target	*Competitor*	*Decoy*
<u>Unhealthy choice set</u>	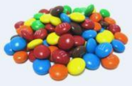<br>M&M's (choco flavour)<br>Taste rating: 8,0<br>Quality of ingredients: 30	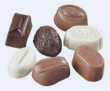<br>Bonbons<br>Taste rating: 6,0<br>Quality of ingredients: 40	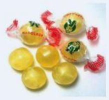<br>Sweets<br>Taste rating: 8,0<br>Quality of ingredients: 20
<u>Healthy choice set</u>	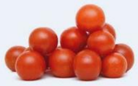<br>Snack tomatoes<br>Taste rating: 6,0<br>Quality of ingredients: 40	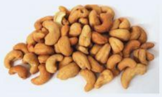<br>Unsalted cashews<br>Taste rating: 8,0<br>Quality of ingredients: 30	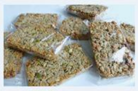<br>Granola cookies<br>Taste rating: 6,0<br>Quality of ingredients: 20
<u>Mixed choice set</u>	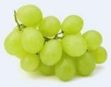<br>White grapes<br>Taste rating: 6,0<br>Quality of ingredients: 40	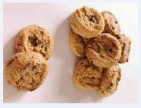<br>Chocolate Chip cookies<br>Taste rating: 8,0<br>Quality of ingredients: 30	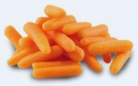<br>Carrots<br>Taste rating: 6,0<br>Quality of ingredients: 20

Source information of the pictures:

M&M's (choco flavour): DocStorage. M7MBerg [Image on the internet]. 2019 [cited 2021 Oct 25]. Available from: https://www.docstorage.nl/docstorage-nieuws/gratis-mms/. Bonbons: picture is taken by first author. Sweets: Le diable@gueur. Le bonbon Napoléon [Image on the internet]. 2014 [cited 2021 Oct 25]. Available from: https://lediablogueur.blogspot.com/2014/06/la-belgique-gourmande.html. Snack tomatoes: Aldi. Cherrytomaten [Image on the internet]. Publication date or year unknown [cited 2021 Oct 25]. Available from: https://www.aldi.nl/producten/aardappels-groente-fruit/groenten/cherrytomaten-6232-1-0.article.html. Unsalted cashews: Noten & Zo. [Image on the internet]. Publication date or year unknown [cited 2021 Oct 25]. Available from: https://www.notenenzo.nl/blog/schijnvrucht. Marloes/Optima Vita. Granola-repen3 [Image on the internet]. 2015 [cited 2021 Oct 25]. Available from: https://www.optimavita.nl/voeding/review-granola-repen-van-de-aldi/. Beste.nl. Witte-druiven-proef [Image on the internet]. 2016 [cited 2021 Oct 25]. Available from: https://www.beste.nl/20299942/deen-witte-druiven-bak-400-gram-of-snoeptomaten-beker-200-gram-voor-euro1-50/. Chocolate Chip cookies: picture is taken by first author. Carrots: Capital City Fruit. Carrots Baby [Image of the internet]. Publication date or year unknown [cited 2021 Oct 25]. Available from: https://capitalcityfruit.com/shop/carrots-baby-1-lb/.

24
